# A cross-national perspective of migration and cancer: incidence of five major cancer types among resettlers from the former Soviet Union in Germany and ethnic Germans in Russia

**DOI:** 10.1186/s12885-019-6058-6

**Published:** 2019-09-02

**Authors:** Philipp Jaehn, Simone Kaucher, Lidia V. Pikalova, Sofia Mazeina, Hiltraud Kajüter, Heiko Becher, Mikhail Valkov, Volker Winkler

**Affiliations:** 1 Brandenburg Medical School Theodor Fontane, Institute of Social Medicine and Epidemiology, Hochstraße 15, Brandenburg an der Havel, 14770 Germany; 2 University Hospital Heidelberg, Institute of Global Health, Im Neuenheimer Feld 324, 69120 Heidelberg, Germany; 3 Tomsk Regional Oncological Hospital, Tomsk Regional Cancer Registry, Lenin str. 115, 634050 Tomsk, Russian Federation; 4 Federal Cancer Registry of North Rhine-Westphalia, Gesundheitscampus 1, 44801 Bochum, Germany; 5 Institute of Medical Biometry and Epidemiology, University Medical Center Hamburg-Eppendorf, Building W34, Martinistraße 52, 20246 Hamburg, Germany; 6 Department of Radiology, Radiotherapy and Oncology, Northern State Medical University, Trotsky av. 51, Arkhangelsk, Russia

**Keywords:** Migrants, Germany, Russia, Cancer, Epidemiology, Health transition

## Abstract

**Background:**

Few studies compared cancer incidence among migrants both to their host countries and to their population of origin. We aimed to compare cancer incidence of ethnic Germans who migrated from the former Soviet Union to Germany (resettlers) to those living in Russia as well as to the German and the Russian general populations.

**Methods:**

The cancer registry of North Rhine-Westphalia identified incident cases of stomach, colorectal, lung, breast and prostate cancer in resettlers and the general population of the administrative district of Münster (Germany) between 2004 and 2013. The Tomsk cancer registry collected the same data in ethnic Germans and the general population of the Tomsk region (Russia). We used standardised incidence rate ratios (SIRs) to compare rates of resettlers and ethnic Germans with the respective general populations.

**Results:**

The total number of person-years under risk was 83,289 for ethnic Germans, 8,006,775 for the population of Tomsk, 219,604 for resettlers, and 20,516,782 for the population of Münster. Incidence of the five investigated cancer types among ethnic Germans did not differ from incidence of the general population of Tomsk. Compared to the general population of Tomsk, incidence among resettlers was higher for colorectal cancer in both sexes (females: SIR 1.45 [95% CI 1.14–1.83], males: SIR 1.56 [95% CI 1.23–1.97]), breast cancer in females (SIR 1.65 [95% CI 1.40–1.95]), and prostate cancer (SIR 1.64 [95% CI 1.34–2.01]). Incidence rates of these cancer types among resettlers were more similar to rates of the general population of Münster. Incidence of stomach and lung cancer did not differ between resettlers and the general population of Tomsk.

**Conclusions:**

After an average stay of 15 years, we observed that incidence of colorectal, breast and prostate cancer among resettlers converged to levels of the general population of Münster. Resettler’s incidence of stomach and lung cancer, however, was comparable to incidence in their population of origin. Causes must be investigated in subsequent analytical studies.

**Electronic supplementary material:**

The online version of this article (10.1186/s12885-019-6058-6) contains supplementary material, which is available to authorized users.

## Background

After guest workers who immigrated to Germany mainly from Italy and Turkey, ethnic German resettlers were the second largest migrant group in Germany in 2017 with around 2.9 million people [1]. Their majority immigrated in the early 1990s from countries of the former Soviet Union with very few formal restrictions [2]. With subsequent introduction of legal restrictions, numbers of immigrants decreased rapidly from 1995 onwards [2, 3]. In 2017, immigration numbers of resettlers in Germany increased for the first time since 2001 with a total of 7000 new registrations [4]. To date, about 400,000 people considering themselves as ethnic Germans still live in Russia according to the 2010 census of the Russian Federation [5]. It had been hypothesised that resettlers imported a high burden of cardiovascular diseases and cancer due to a high exposure to risk factors and low socioeconomic status in countries of the former Soviet Union [6]. Regarding cancer, this hypothesis was not confirmed, when studies found that overall cancer mortality among male resettlers was similar compared to the general German population, and lower among female resettlers. However, mortality ratios showed site-specific cancer differences [7]. Focusing on cancer incidence in comparison to the respective regional populations in Germany, female resettlers had lowered overall, colorectal, lung and breast cancer incidence, while male resettlers showed lowered overall and prostate cancer incidence, but elevated stomach cancer incidence [8, 9]. Furthermore, overall cancer incidence converged since 1994 reaching levels of the general German population in 2013 [8]. Finally, higher odds of having an advanced tumour stage at diagnosis among resettlers compared to the general population are in line with findings of lower health care utilisation of this minority group [8, 10–12].

Of all cancer types, incidence rates in Russia in 2018 were highest for breast cancer among females and lung cancer among males, while in Germany breast cancer showed the highest incidence among females and prostate cancer among males [13]. For all cancer types combined, age-adjusted incidence increased globally between 1990 and 2015, however, more rapidly in Germany compared to Russia [14]. In both Russia and Germany in 2018, mortality rates were highest for lung cancer among males and breast cancer among females [15].

The observation of cancer incidence trends among migrant populations is a unique opportunity to add evidence to our understanding of cancer aetiology [16]. Although reasons of changes in cancer incidence rates among migrant populations are poorly understood, past analyses have highlighted the importance of the changing physical and social environment on cancer risk [17]. A summary of cancer incidence among non-western migrants in Europe demonstrated that most migrant populations show lower overall cancer incidence, higher incidence of cervical and stomach cancer and lower incidence of colorectal, breast and prostate cancer compared to general populations of their host countries [18].

Several hypotheses have been proposed to explain migrant’s overall favourable health, which has been extensively documented using mortality data [19]. The widely cited healthy migrant effect assumes that only healthy people migrate and, hence, the migration process describes a form of selection phenomenon. Others have argued that migrants might return to their countries of origin when they fall ill (salmon effect), that living in segregated communities protects from health deterioration (ethnic enclave hypothesis), or that migrants profit from immediate effects of a better healthcare system in the destination country (health transition hypothesis) [20]. Convergence theory states that interaction of migrants with the host population leads to an adoption of health-related behaviours and consequently migrant’s health profile converges to that of the host population [20].

It is evident that the majority of studies on migration and health have put findings in context with the new host population. Comparisons to countries of origin or to the specific populations of origin are scarce. A framework of migrant’s health, which has been derived from the sociological literature suggests intensifying cross-national research in public health in order to understand the health of migrant populations as a result of health in both sending and receiving countries [21].

To our knowledge, there is no study investigating any health outcome among ethnic Germans in Russia, or comparing health outcomes cross-nationally between ethnic Germans that remained in Russia, resettlers in Germany and the corresponding majority populations of both countries.

## Methods

The aim of this study was to apply a cross-national framework to compare cancer incidence of five major cancer sites among *resettlers* living in the administrative district (AD) of Münster in Germany to *ethnic Germans* living in the region of Tomsk (Tomsk Oblast) in Russia and to both regional general populations.

### Cancer cases and population data of resettlers and the general population of the AD Münster

Cancer incidence of resettlers in Germany was determined in a historical registry-based cohort called the AMIN-study [8]. The AMIN-cohort of 32,972 resettlers from countries of the former Soviet Union who immigrated to the AD Münster between 1990 and 2001 was set up by identifying resettlers in historical immigration records of the registry offices in local municipalities. The sample can be considered representative of all resettlers in the AD Münster including 52.9% of resettlers who were originally registered in this region. Incident cancer cases of the AMIN-study were identified by the federal cancer registry of North Rhine-Westphalia (NRW) based on a pseudonymised record linkage [22, 23]. Encrypted personal identifiers of each cohort member were used to identify incident cases of resettlers [8]. Person time at risk of resettlers was approximated using date of immigration and date of diagnosis or the 31st of December 2013 as end of follow-up while estimating mortality and out-migration from a previous cohort study [24].

The NRW cancer registry also provided cancer cases and yearly midyear population figures of the general population of the AD Münster, therefore, cancer incidence rates of the general population of Münster could be estimated.

### Cancer cases and population data of ethnic Germans and the general population of the Tomsk Oblast

To date, about 10,000 ethnic Germans reside in the Tomsk Oblast, Russia [5]. The Tomsk cancer registry collects information on self-reported nationality for each cancer diagnosis. The variable self-reported nationality contained no missing data. Based on this information all cancer diagnoses of ethnic Germans were extracted from the registry.

To estimate person time at risk of ethnic Germans, we used population figures of the Russian population census, which reported German ethnicity based on the self-reported categories “German”, “German Mennonite”, and “other German”, where “Mennonite” refers to German members of a protestant free church [25]. Age group- and sex-specific population figures of ethnic Germans in Tomsk were available for the years 2002 and 2010 [5, 26]. Midyear population counts for the years 2004 to 2013 were derived by assuming a linear sex- and age group-specific change from 2002 to 2010, which was then extrapolated to 2013. Because we have no data that supports the assumption of a linear trend, three scenarios of population development among ethnic Germans in Tomsk were applied in a sensitivity analysis to examine robustness of results to different assumptions about the population development. The three scenarios considered were a constant mean population, two periods with constant population, and a linear change until 2010 followed by a constant population after 2010.

Age group- and sex-specific incident cancer cases for the general population of the Tomsk Oblast were provided by the Tomsk cancer registry. They also provided the annual age group- and sex-specific midyear population counts.

### Processing of cancer registry data

We analysed cancer incidence between 2004 and 2013 for the following sites: stomach (C16.0–9), colorectum (C18.0–21.8), bronchus and lung (C34.0–9), female breast (C50.0–9) and prostate (C61). These are the most common cancer types among resettlers [8]. Information on date of diagnosis, ICD-O-3 M code, sex, and age was provided. We selected only cases with primary malignant behaviour (ICD-O-3 M code, 5th digit: 3), multiple primaries were determined according to rules of the International Agency for Research on Cancer (IARC) (following recommendation 2: “Cancers which occur in any 4th character subcategory of colon (C18) and skin (C44) should be registered as multiple primary cancers.”) [27]. Cases registered on the basis of death certificate only (DCO) were included into analysis.

The datasets of the Münster and Tomsk cancer registry were checked for data validity using the IARC/IACR crg Tool [28]. The tool indicates cases of missing data, unlikely diagnoses, unlikely combinations of tumour characteristics and multiple primary cancers. Two records were removed from the Tomsk dataset; one due to a missing ID and one due to missing age. There were no implausible diagnoses in the data of the Münster cancer registry.

### Statistical methods

All statistical analyses except the calculation of directly age-standardised rates were restricted to ages over 20. Age-standardised rates were not restricted to ages over 20 to enable comparisons with routinely reported age-standardised rates. Age group-specific rates were displayed for each cancer site in each population.

We calculated age-standardised rates (ASRs) per 100,000 person-years for the period 2004–2008 and 2009–2013 among the general populations of Tomsk and Münster. Rates were standardised against the old world standard (Segi, modified by Doll) [29]. Standardised incidence rate ratios (SIRs) were calculated for ethnic Germans in Tomsk and resettlers in Germany compared to both standard populations namely the general populations of the AD Münster and of the Tomsk Oblast. To calculate SIRs, age was categorised in 5 year age groups, starting from the age of 20 until the age of 70. Ages over 70 were grouped together, because this age group was only available in aggregated format for ethnic Germans. The expected number of cases was calculated multiplying the sex-, age group- and calendar year-specific person-years (resettlers) or the midyear populations (ethnic Germans, Tomsk) with the respective rates of the standard population. Additionally, the general population of Tomsk was standardised to the Münster general population. Exact 95% confidence intervals (CIs) of SIRs and ASRs were calculated [30, 31]. Analysis was done using R version 3.5.1.

## Results

During the study period from 2004 to 2013, annual person-years were on average 8329 among ethnic Germans, 800,678 among the general population of Tomsk, 21,960 among resettlers and 2,051,678 among the general population of Münster. Numbers of cases and person-years stratified by sex and time period are displayed in Table 1.

**Table 1:** Number of cases and person-years among ethnic Germans, resettlers and the background populations (from age 20)

		Tomsk population		Ethnic Germans		Resettlers		Münster population	
		N	%	N	%	N	%	N	%
Cancer cases									
Stomach cancer	Female	1174	43.2	9	31.0	20	37.0	2191	41.4
	Male	1542	56.8	20	69.0	34	63.0	3100	58.6
	2004–2008	1370	50.4	16	55.2	16	29.6	2690	50.8
	2009–2013	1346	49.6	13	44.8	38	70.4	2601	49.2
	Total	2716	100.0	29	100.0	54	100.0	5291	100.0
Colorectal cancer	Female	1986	55.9	19	51.4	69	49.6	10,903	47.9
	Male	1565	44.1	18	48.6	70	50.4	11,841	52.1
	2004–2008	1627	45.8	23	62.2	59	42.4	11,626	51.1
	2009–2013	1924	54.2	14	37.8	80	57.6	11,118	48.9
	Total	3551	100.0	37	100.0	139	100.0	22,744	100.0
Lung cancer	Female	811	19.1	8	13.8	18	14.6	6034	31.4
	Male	3431	80.9	50	86.2	105	85.4	13,194	68.6
	2004–2008	2053	48.4	36	62.1	47	38.2	9211	47.9
	2009–2013	2189	51.6	22	37.9	76	61.8	10,017	52.1
	Total	4242	100.0	58	100.0	123	100.0	19,228	100.0
Breast cancer	Female	3495	100.0	48	100.0	140	100.0	22,777	100.0
	2004–2008	1576	44.9	23	47.9	60	42.9	11,129	48.9
	2009–2013	1919	55.1	25	52.2	80	57.1	11,648	51.1
	Total	3495	100.0	48	100.0	140	100.0	22,777	100.0
Prostate cancer	Male	1915	100.0	24	100.0	94	100.0	21,060	100.0
	2004–2008	693	36.2	9	37.5	31	33.0	10,483	49.8
	2009–2013	1222	63.8	15	62.5	63	67.0	10,577	50.2
	Total	1915	100.0	24	100.0	94	100.0	21,060	100.0
Person-years	Female	4,338,787	54.2	44,387	53.3	114,544	52.2	10,634,958	51.8
	Male	3,666,556	45.8	38,903	46.7	105,060	47.8	9,881,824	48.2
	2004–2008	3,905,135	48.8	45,984	55.2	107,075	48.8	10,211,614	49.8
	2009–2013	4,100,207	51.2	37,305	44.8	112,528	51.2	10,305,168	50.2
	Total	8,006,775	100.0	83,289	100.0	219,604	100.0	20,516,782	100.0

**Fig. 1:**
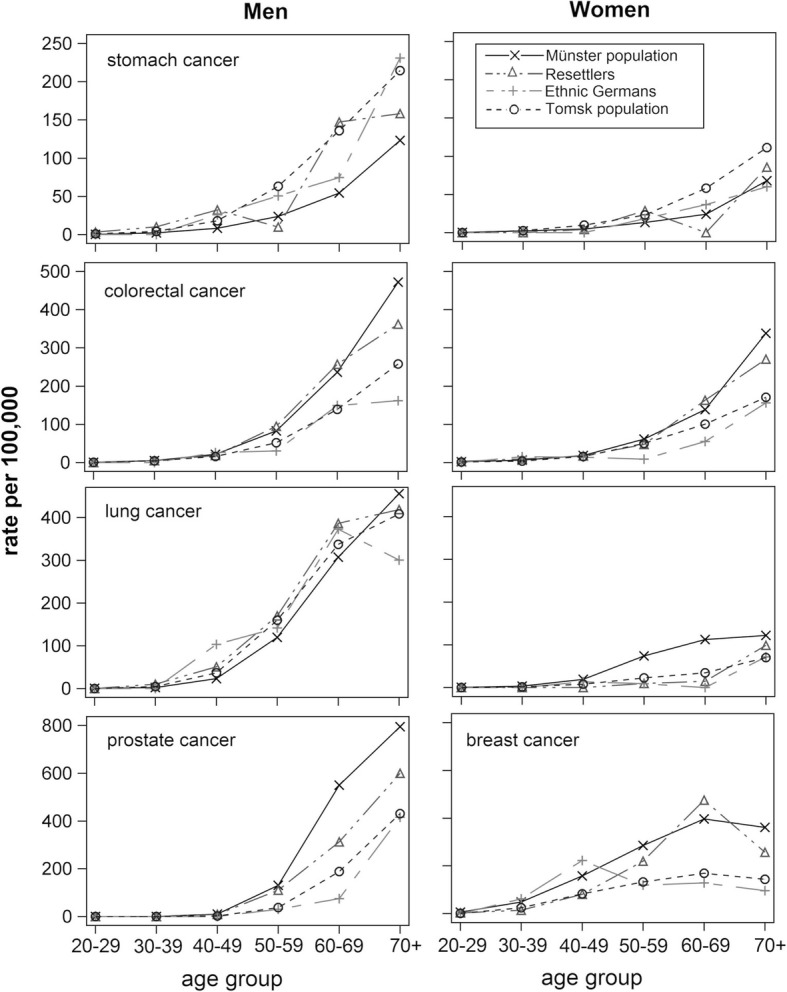
Age group-specific rates among ethnic Germans, resettlers and the respective background populations (from age 20)

Age-specific rates of lung cancer among female ethnic Germans and female resettlers were similar to the female population of Tomsk (Fig. 1). Breast cancer rates among ethnic Germans were similar to rates of the general population of Tomsk, however, among resettlers, they were similar to rates of the general population of Münster. Male resettlers’ age-specific rates of prostate cancer was in between rates of the general populations of Tomsk and Münster, while those of ethnic German’s were similar to rates in the general population of Tomsk.

ASRs in females and males of the general population of Tomsk and Münster in the periods of 2004–2008 and 2009–2013 are displayed in Table 2. ASRs of stomach cancer were 24.9 per 100,000 (95% CI 23.1–26.9) among males in Tomsk and 11.4 per 100,000 (95% CI 10.8–12.1) among males in Münster in the period 2009–2013. ASRs of female lung cancer were 8.6 per 100,000 (95% CI 7.7–9.6) in Tomsk and 24.3 per 100,000 (95% CI 23.4–25.3) in Münster in the same period. Finally, the ASR of female breast cancer was 45.9 per 100,000 (95% CI 43.8–48.2) in Tomsk and 93.9 per 100,000 (95% CI 92.0–95.8) in Münster between 2009 and 2013.

**Table 2:** Age-standardised incidence rates of the populations of Tomsk and Münster (from age 0)

		Tomsk population		Münster population	
		2004–2008	2009–2013	2004–2008	2009–2013
		ASR (95% CI)	ASR (95% CI)	ASR (95% CI)	ASR (95% CI)
Cancer	Sex				
Stomach cancer	Female	12.2 (11.1–13.4)	11.8 (10.8–13.0)	6.4 (6.0–6.9)	5.9 (5.4–6.3)
	Male	28.4 (26.4–30.6)	24.9 (23.1–26.9)	12.7 (12.1–13.4)	11.4 (10.8–12.1)
Colorectal cancer	Female	19.8 (18.5–21.4)	21.6 (20.3–23.1)	32.4 (31.4–33.4)	29.0 (28.0–30.0)
	Male	25.4 (23.5–27.5)	29.4 (27.4–31.6)	47.8 (46.5–49.1)	43.5 (42.3–44.7)
Lung cancer	Female	8.5 (7.6–9.5)	8.6 (7.7–9.6)	20.0 (19.2–20.9)	24.3 (23.4–25.3)
	Male	59.3 (56.4–62.4)	58.5 (55.7–61.4)	54.6 (53.2–56.1)	51.4 (50.1–52.7)
Breast cancer	Female	39.5 (37.4–41.6)	45.9 (43.8–48.2)	93.1 (91.2–95.0)	93.9 (92.0–95.8)
Prostate cancer	Male	25.9 (23.9–28.0)	42.0 (39.6–44.6)	84.5 (82.8–86.3)	80.1 (78.4–81.7)

There was some evidence that the incidence of colorectal, breast and prostate cancer among resettlers was higher compared to the general population of Tomsk (Table 3). Incidence among ethnic Germans did not differ from incidence in the general population of Tomsk, all 95% CIs included one.

**Table 3:** Standardised incidence rate ratios of ethnic Germans and resettlers compared to the background populations (from age 20)

		compared to the Tomsk population		compared to the Münster population		
		Ethnic Germans	Resettlers	Tomsk population	Ethnic Germans	Resettlers
		SIR (95% CI)	SIR (95% CI)	SIR (95% CI)	SIR (95% CI)	SIR (95% CI)
Cancer cases	Sex					
Stomach cancer	Female	0.57 (0.30–1.10)	0.70 (0.45–1.08)	1.80 (1.70–1.91)	1.01 (0.52–1.94)	1.23 (0.80–1.91)
	Male	0.88 (0.57–1.37)	0.78 (0.55–1.09)	2.20 (2.09–2.31)	1.88 (1.21–2.92)	1.64 (1.19–2.32)
Colorectal cancer	Female	0.72 (0.46–1.13)	1.45 (1.14–1.83)	0.62 (0.59–0.65)	0.43 (0.28–0.68)	0.88 (0.69–1.11)
	Male	0.78 (0.49–1.24)	1.56 (1.23–1.97)	0.60 (0.57–0.63)	0.46 (0.29–0.72)	0.92 (0.73–1.16)
Lung cancer	Female	0.73 (0.37–1.46)	0.91 (0.57–1.44)	0.41 (0.38–0.44)	0.31 (0.15–0.61)	0.38 (0.24–0.61)
	Male	1.00 (0.76–1.32)	1.10 (0.91–1.33)	1.13 (1.09–1.16)	1.10 (0.83–1.45)	1.23 (1.02–1.49)
Breast cancer	Female	1.12 (0.85–1.49)	1.65 (1.40–1.95)	0.45 (0.43–0.46)	0.50 (0.37–0.66)	0.74 (0.63–0.87)
Prostate cancer	Male	0.83 (0.56–1.24)	1.64 (1.34–2.01)	0.42 (0.40–0.44)	0.35 (0.24–0.52)	0.75 (0.61–0.91)

*SIR:* Standardised incidence rate ratio of age groups over 20 years

*95% CI:* 95% confidence interval

Compared to the male population of Münster, stomach cancer incidence was elevated among male ethnic Germans from Tomsk, CIs did not include one (Table 3). On the other hand, there was some evidence that incidence of colorectal cancer among both sexes, female lung cancer, breast and prostate cancer among ethnic Germans from Tomsk were lower compared to the general population of Münster. Male resettlers showed evidence for higher incidence of stomach and lung cancer compared to the male population of Münster. Furthermore, incidence of female lung, female breast and prostate cancer was lower among resettlers compared to the Münster general population and CIs did not include one. SIRs did not change considerably when applying the alternative three scenarios of population development among ethnic Germans in Tomsk (see Additional file 1). In all three scenarios, stomach cancer incidence among male ethnic Germans from Tomsk was elevated compared to the male population of Münster. Finally, colorectal cancer among both sexes, female lung cancer, breast and prostate cancer incidence were lower compared to the general population of Münster. All other rates did not differ from the general population of Münster in any scenario.

## Discussion

Ethnic Germans in Tomsk showed similar age-standardised incidence of all studied cancer types in comparison to the general population of Tomsk. Approximately 15 years after the migration of ethnic German resettlers from the former Soviet Union to Germany, a transition of cancer incidence was observed [3]. Among resettlers, incidence of colorectal, breast and prostate cancer was more similar to levels of the general population of Münster and differed from incidence among ethnic Germans in Tomsk. Their incidence rates of stomach and lung cancer were comparable to levels found among a population of ethnic Germans in Russia.

The five investigated cancer types comprise a considerable share of cancer burden in both countries. The estimated proportions of incident stomach, colorectal, lung, breast and prostate cancer cases compared to all cancer cases was 52% in Russia and 55% in Germany [32].

Findings of cancer incidence among resettlers in the Münster cohort have reproduced earlier findings among resettlers in the Saarland [9, 33]. Cancer incidence among resettlers furthermore mirrors incidence patterns of cancers of the respiratory system, breast and prostate among Turkish immigrants in Germany and generally non-western migrants in Europe [18, 34]. Spallek and colleagues investigated cancer mortality among Turkish immigrants in Europe from a cross-national point of view [35]. They found that breast and stomach cancer mortality was in between levels of the host countries and Turkey [35]. Causes of mortality rates among Turkish immigrants may, however, differ from resettlers, as for example dietary patterns among adolescent Russian Germans differed from adolescent immigrants from Turkey [36].

Data of the Tomsk cancer registry was recently used in the CONCORD-3 study [37]. Hence, we consider the data quality of the Tomsk cancer registry to be acceptable. Completeness of cancer registration in the AD of Münster is considered to be high since 1994 and further improved since then. In addition, cancer registration became mandatory by law in 2005 [38].

A limitation of this study was the lack of an individual follow-up of ethnic Germans in Tomsk. Therefore, census data were used to approximate rate denominators. Results assuming three alternative scenarios of population development using these census data did not lead to different conclusions. Furthermore, statistical power to detect differences between ethnic Germans and the general populations of Tomsk or Münster was low for rare cancers like stomach cancer and moderate for more frequent types.

Information bias could have occurred, because data on ethnicity was self-reported in the Russian census and the Tomsk cancer registry data. On the other hand, self-reported ethnicity might be an adequate indicator as people strongly identifying themselves as German might be more likely to report a German ethnicity. Finally, incident cancer cases among ethnic Germans in Tomsk might have been under registered because calculated SIRs in comparison to the general population of Tomsk were below 1 for most cancer types.

Considering resettlers in the AD Münster, our estimation method of person time is based on out-migration rates, which are supposed to be higher among younger age groups. Thus, the procedure described in [24] assumes higher loss-to-follow up among young resettlers (< 30 years of age) compared to older ones. As resettlers mostly immigrated in younger ages (mean age: 29.1 years of age), the estimation procedure may have led to an underestimation of person-years of the AMIN-study and therefore, incidence of resettlers might have been overestimated. However, sensitivity analyses showed only slightly deviating results [9].

Finally, incidence rates of breast cancer among resettlers and the general population in the AD Münster are driven by the introduction of the mammography screening programme (MSP) in 2005 [39]. In 2008, an incidence peak was described before rates stabilised again in 2010 [39]. This might have exaggerated differences of breast cancer incidence between populations in Germany and Russia during the study period.

Having these considerations in mind, differences of incidence between resettlers and ethnic Germans in Russia might have been overestimated. However, as differences of SIRs of colorectal and prostate cancer between ethnic Germans in Russia and resettlers in Germany are large, we consider our findings to be robust. Differences of breast cancer incidence, however, might have been driven by the introduction of the MSP in Germany.

The population of origin of resettlers in Germany are ethnic Germans from former Soviet Union countries. We approximated this population by ethnic Germans living in the Tomsk Oblast, Russia. Resettlers living in Germany come mostly from Kazakhstan and Russia [2]. Therefore, the location of Tomsk close to the Kazakh border might be a good approximation of the population of origin of most resettlers. Ethnic Germans in Russia, furthermore, share the same ancestors who emigrated to the Russian empire in the 18th and 19th century. The German minority lived there until around World War One in relatively closed and privileged communities. Thereafter they faced increasing discrimination and were deported to Kazakhstan and Siberia during the 1930s [40]. After the Second World War until 1990, ethnic Germans have been oppressed by the Soviet regime [40].

The migration process observed in this study has some unique features. First, it is a “back-migration” of a population after about 200–300 years. Second, major restrictions during the migration process were absent. In fact, when most resettlers migrated to Germany during the early 1990s, they were invited to Germany [2]. Therefore, the migration process is different from for example work migration, where one would expect a selection towards young and healthy individuals [20].

Early detection programmes for colorectal, breast and prostate cancer, but not for stomach and lung cancer are covered by the statutory health insurance in Germany [41]. In conclusion, we hypothesise that differences in incidence of colorectal, breast and prostate cancer between resettlers and their population of origin might be due to early detection and screening measures which were accessible to resettlers in Germany. Resettlers obtained the German citizenship immediately after immigration [2]. Furthermore, self-rated language proficiency among resettlers was higher compared to other migrant groups in Germany [2]. It is important to stress, however, that our results do not conflict with earlier studies indicating less preferable health seeking behaviour and advanced stage at diagnosis among resettlers compared to the general German population [10, 11].

Our results support the hypothesis that direct exposure to the health care system of the host country might have had immediate effects on cancer incidence of resettlers [20]. An acculturation of behavioural risk factors has been described for smoking among resettlers in Germany [42]. However, we have observed that lung cancer incidence among both female and male resettlers was similar compared to rates in their country of origin. Hence, we hypothesise that changes in lifestyle do not yet impact on cancer incidence rates of resettlers.

Applying a cross-national perspective, our findings support the hypothesis that both the environment in the country of origin and in the country of destination shape the health of migrants [20, 21]. It would be worthwhile to study whether contacts of ethnic Germans in Tomsk to resettlers in Germany influence health of both populations like suggested by the cross-national framework of migration and health [21].

## Conclusion

In conclusion, results of our study suggest that incidence rates of cancer types for which measures of early detection were available converged to rates among the general German population. We reproduced the finding that cancer incidence among migrant populations often mirrors states in between the population of origin and the new host population. Future research should determine risk factors of cancer incidence among migrant populations in order to create more solid evidence. Finally, more evidence on cross-national ties of migrant populations is needed in order to investigate international bounds and their effects on health.

## Additional file

Additional file 1: Sensitivity analysis of standardised incidence rate ratios of ethnic Germans living in Tomsk assuming three different scenarios of underlying population figures. (DOCX 31 kb)

## Data Availability

The datasets used and/or analysed during the current study are available from V. Winkler subject to collaboration agreement.

## Abbreviations

AD: administrative district
ASR: age-standardised rate
CI: confidence interval
IARC: International Agency for Research on Cancer
ICD-O M: International Classification of Diseases for Oncology, morphology code
MSP: mammography screening programme
NRW: North Rhine-Westphalia
SIR: standardised incidence rate ratio
