# Supplementary material for: A cross-national perspective of migration and cancer: incidence of five major cancer types among resettlers from the former Soviet Union in Germany and ethnic Germans in Russia
Source: BMC Cancer. 2019 Sep 2;19:869. doi: 10.1186/s12885-019-6058-6 (PMC6721094; doi:10.1186/s12885-019-6058-6)
Supplement: Supplementary file 1 — Sensitivity analysis of standardised incidence rate ratios of ethnic Germans living in Tomsk assuming three different scenarios of underlying population figures. (DOCX 31 kb) [file 12885_2019_6058_MOESM1_ESM.docx]

**Additional file 1**

**Title:**

Sensitivity analysis of standardised incidence rate ratios of ethnic Germans living in Tomsk assuming three different scenarios of underlying population figures

**Description:**

Sensitivity analysis:

Scenario 1: The annual sex- and age group-specific population of ethnic Germans living in Tomsk was assumed to be the arithmetic mean of the population of 2002 and 2010.

Scenario 2: The annual sex- and age group-specific population of ethnic Germans living in Tomsk was assumed to be the population of 2002 in the period 2004-2006 and the population of 2010 in the period 2007-2013.

Scenario 3: The annual sex- and age group-specific population of ethnic Germans living in Tomsk was assumed to follow a linear sex- and age group-specific change from 2002 to 2010 and to be the population of 2010 during the period 2010-2013.

**Additional Table: Standardised incidence rate ratios of ethnic Germans compared to the general populations of Tomsk and Münster (assuming scenarios 1,2 and 3)**

|  |  |  | **Scenario 1** | | **Scenario 2** | | | **Scenario 3** | |
| --- | --- | --- | --- | --- | --- | --- | --- | --- | --- |
|  |  |  | compared to Tomsk | compared to Münster | compared to Tomsk | | compared to Münster | compared to Tomsk | compared to Münster |
|  |  |  |  |  |  | |  |  |  |
| cancer | sex |  | SIR (95% CI) | SIR (95% CI) | SIR (95% CI) | | SIR (95% CI) | SIR (95% CI) | SIR (95% CI) |
|  |  |  |  |  |  | |  |  |  |
| stomach cancer | females |  | 0.55 (0.27-1.16) | 0.98 (0.51-1.89) | 0.57 (0.29-1.09) | | 1.00 (0.52-1.92) | 0.57 (0.30-1.09) | 1.00 (0.52-1.93) |
|  | males |  | 0.86 (0.55-1.33) | 1.82 (1.17-2.82) | 0.87 (0.56-1.35) | | 1.86 (1.21-2.89) | 0.88 (0.57-1.36) | 1.87 (1.20-2.89) |
|  |  |  |  |  |  | |  |  |  |
| colorectal cancer | females |  | 0.70 (0.45-1.10) | 0.42 (0.27-0.66) | 0.72 (0.46-1.12) | | 0.43 (0.27-0.67) | 0.72 (0.46-1.12) | 0.43 (0.27-0.67) |
|  | males |  | 0.75 (0.47-1.18) | 0.44 (0.28-0.69) | 0.78 (0.49-1.23) | | 0.45 (0.28-0.71) | 0.77 (0.49-1.23) | 0.45 (0.28-0.72) |
|  |  |  |  |  |  | |  |  |  |
| lung cancer | females |  | 0.71 (0.36-1.42) | 0.30 (0.15-0.60) | 0.72 (0.36-1.45) | | 0.30 (0.15-0.61) | 0.73 (0.36-1.45) | 0.31 (0.15-0.61) |
|  | males |  | 0.98 (0.74-1.29) | 1.07 (0.81-1.41) | 0.99 (0.75-1.31) | | 1.09 (0.82-1.44) | 0.99 (0.76-1.32) | 1.09 (0.83-1.44) |
|  |  |  |  |  |  | |  |  |  |
| breast cancer | females |  | 1.06 (0.80-1.41) | 0.47 (0.35-0.62) | 1.11 (0.83-1.47) | | 0.49 (0.37-0.65) | 1.11 (0.83-1.47) | 0.49 (0.37-0.65) |
|  |  |  |  |  |  | |  |  |  |
| prostate cancer | males |  | 0.79 (0.53-1.18) | 0.34 (0.23-0.50) | 0.81 (0.54-1.21) | | 0.35 (0.23-0.52) | 0.82 (0.55-1.22) | 0.35 (0.23-0.52) |
|  |  |  |  |  |  | |  |  |  |
| SIR: Standardised incidence rate ratio of age groups over 20 years | | | | | |  |  |  |  |
| 95% CI: 95% confidence interval | | | | | |  |  |  |  |
